# Divergence of metabolites in three phylogenetically close *Monascus* species (*M. pilosus*, *M. ruber,* and *M. purpureus*) based on secondary metabolite biosynthetic gene clusters

**DOI:** 10.1186/s12864-020-06864-9

**Published:** 2020-10-01

**Authors:** Yuki Higa, Young-Soo Kim, Md. Altaf-Ul-Amin, Ming Huang, Naoaki Ono, Shigehiko Kanaya

**Affiliations:** 1 R&D Center, Kobayashi Pharmaceutical Co., Ltd, Ibaraki-shi, Toyokawa, 1-30-3, Osaka, Japan; 2 Graduate School of Science and Technology, Nara Institute of Science and Technology, Ikoma-shi, Takayama-cho, Nara, 8916-5 Japan; 3 Data Science Center, Nara Institute of Science and Technology, Ikoma-shi, Takayama-cho, Nara, 8916-5 Japan

**Keywords:** *Monascus* species, Comparative genomics, Polyketide synthase, Monacolin K, Citrinin, Fermentation, Natural pigment, Food microbiology

## Abstract

**Background:**

Species of the genus *Monascus* are considered to be economically important and have been widely used in the production of yellow and red food colorants. In particular, three *Monascus* species, namely, *M. pilosus*, *M. purpureus*, and *M. ruber*, are used for food fermentation in the cuisine of East Asian countries such as China, Japan, and Korea. These species have also been utilized in the production of various kinds of natural pigments. However, there is a paucity of information on the genomes and secondary metabolites of these strains. Here, we report the genomic analysis and secondary metabolites produced by *M. pilosus* NBRC4520, *M. purpureus* NBRC4478 and *M. ruber* NBRC4483, which are NBRC standard strains. We believe that this report will lead to a better understanding of red yeast rice food.

**Results:**

We examined the diversity of secondary metabolite production in three *Monascus* species (*M. pilosus*, *M. purpureus*, and *M. ruber*) at both the metabolome level by LCMS analysis and at the genome level. Specifically, *M. pilosus* NBRC4520, *M. purpureus* NBRC4478 and *M. ruber* NBRC4483 strains were used in this study. Illumina MiSeq 300 bp paired-end sequencing generated 17 million high-quality short reads in each species, corresponding to 200 times the genome size. We measured the pigments and their related metabolites using LCMS analysis. The colors in the liquid media corresponding to the pigments and their related metabolites produced by the three species were very different from each other. The gene clusters for secondary metabolite biosynthesis of the three *Monascus* species also diverged, confirming that *M. pilosus* and *M. purpureus* are chemotaxonomically different. *M. ruber* has similar biosynthetic and secondary metabolite gene clusters to *M. pilosus*. The comparison of secondary metabolites produced also revealed divergence in the three species.

**Conclusions:**

Our findings are important for improving the utilization of *Monascus* species in the food industry and industrial field. However, in view of food safety, we need to determine if the toxins produced by some *Monascus* strains exist in the genome or in the metabolome.

## Background

Species of the genus *Monascus* are economically important because they have been widely used in the production of yellow and red food colorants. In particular, *M. pilosus*, *M. purpureus*, and *M. ruber* are commonly used for food fermentation in the cuisine of East Asian countries including China, Japan, and Korea [1–3]. These species are also utilized to produce various kinds of natural pigments (reviewed in [4, 5]), including yellow pigments such as ankaflavin, monascin and rubropunctatin, orange pigments such as monascorubrin, purple pigments such as rubropunctamin and monascorubramin, and red pigments such as monascorubramine, *N*-glucosylmonascorubramine, *N*-glucosylrubropunctamine, *N*-glutarylmonascorubramine and *N*-glutarylrubropunctamin. One example is the traditionally fermented rice that contains at least 6 pigments from *Monascus* spp., including rubropunctatin, monascorbrin, rubropunctamin, monascorubramin, ankaflavin, and monascin [6]. The prediction of biosynthetic pathways for structurally diverse azaphilone pigments has recently been reported [7]. Recently, azaphilone pigment has been found to be produced by *Penicillium marneffei* and *Talaromyces atroroseus* [8, 9].

*M. pilosus* is a well-known fungus that produces several bioactive metabolites, such as monacolins K and L, as well as several pigments that are related with biological activities including anti-obesity, regulation of lipid metabolism, and Alzheimer’s disease at the in vitro and in vivo levels [8]. *M. purpureus* contains unsaturated fatty acids, sterols, monacolin and azaphilone pigments. It has been reported that these compounds are effective in lowering cholesterol, as well as in the treatment of diabetes, cardiovascular diseases, and some cancers [10]. *M. ruber* contains monacolin and azaphilone pigment. Recent studies have investigated *M. ruber* as an alternative to nitrite substitutes in meat processing [11].

The complete genome sequence of the industrial strain *M. purpureus* YY-1 is already available [12]. Here, we determined the draft genome sequences of *M. ruber* and *M. pilosus* in order to compare their different phenotypes. Understanding the diversity of the secondary metabolites produced by these species at the genome level is crucial for their industrial applications. We analyzed *M. pilosus* NBRC4520, *M. purpureus* NBRC4478, and *M. ruber* NBRC4483 to determine the diversity of the pigments based on metabolome data and secondary metabolite-related gene clusters such as monacolins, citrinin and azaphilone pigment. Several pigments are synthesized by PKS (poly ketide synthase) enzyme and NRPS (nonribosomal peptide) enzyme systems, which are encoded by large gene clusters in the genome. Comparison of such gene clusters between the three species will provide new insights into the potential production of novel pigments.

*Monascus* species produce a multitude of compounds, including polyketides, unsaturated fatty acids, and phytosterols. Monacolins, especially monacolin K, inhibit 3-hydroxy-3-methylglutaryl-coenzyme A reductase, which is the rate-limiting step in cholesterol biosynthesis. These compounds found in red yeast rice prevent high cholesterol levels that causes atherosclerosis [13]. Hence, it is expected that metabolites related to *Monascus* pigments can contribute to human health. However, citrinin was found as an undesirable contaminant in red yeast rice [14]. Specifically, citrinin has been reported to be nephrotoxic and must be strictly controlled.

Thus, it is important to define the diversity of biosynthetic pathways responsible for secondary metabolite production in economically important species such as *Monascus*. Red yeast rice is used in many foods around the world. Foods using red yeast rice must contain substances such as monacolin K that contribute to health, but must not contain citrinin, which causes nephrotoxicity. Therefore, further studies of genes involved in the synthesis of secondary metabolites by *Monascus* need to be carried out. In the present study, we determined the genome sequences of *M. pilosus*, *M. purpureus*, and *M. ruber.* The phylogenetic and chemotaxonomic differences between these three species were characterized by analyzing the gene clusters associated with secondary metabolites.

## Results

### Cultivation of *Monascus* and LCMS analysis

**Fig. 1:**
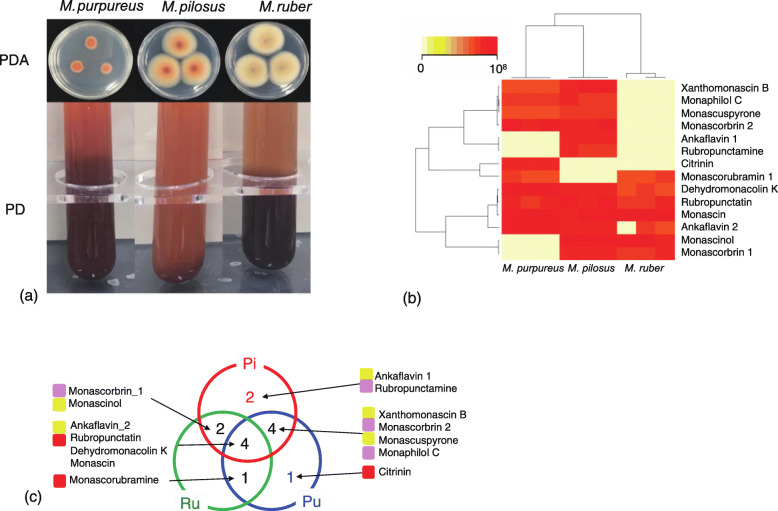
Pigments for three *Monascus* species for PD agar and PDL cultures. **a** *Monascus* species are cultured in PD agar and PDL medium at 30 °C. **b** 2D clustering of pigment quantities among the three species. **c** Venn diagram of the pigments observed in PDL culture. Two-letter abbreviations use used for the Venn diagrams: Pi, *M. pilosus* NBRC 4520; Ru, *M. ruber* NBRC 4483; Pu, *M. purpureus* NBRC 4478

*Monascus* species can produce several types of azaphilones, including nitrogenated azaphilones, N-glucosyl azaphilones, amino acid derivative azaphilones, and citrinins [4]. We cultivated the three *Monascus* species to compare the production of the pigments and their related metabolites using both agar and liquid medium, i.e., potato dextrose agar (PDA) and potato dextrose liquid (PDL) medium, which is the most frequently used culture medium for *Monascus* growth and metabolite production [15]. As shown in Fig. 1a, distinct colony shapes and colors could be observed on PDA and in PDL among *M. pilosus, M. ruber*, and *M. purpureus*. In order to quantify the difference of the pigment contents, we analyzed the medium using LC-MS and identified 14 pigments in total. Figure 1b showed the concentration of 14 metabolites quantified in three biological replicates in the three species as a heatmap with two-dimensional hierarchical clustering to display their similarity. This result confirmed that not all these metabolites are synthesized in the three species. The numbers of pigments commonly identified in each species are summarized as the Venn diagram (Fig. 1c). Dehydromonacolin K, rubropunctatin, monascin, and ankaflavin 2 were commonly produced by all three *Monascus* species. Of the three species, *M. pilosus* produced the greatest number of pigments (12 in total; Fig. 1c). Ten pigments, except monascorubramine, were produced by *M. pilosus,* while ankaflavin 1 and rubropunctamine were only produced by *M. pilosus*. Citrinin, a mycotoxin with nephrotoxic activity in mammals [16], was only produced by *M. purpureus*.

### Biosynthetic pathway of detected metabolites

**Fig. 2:**
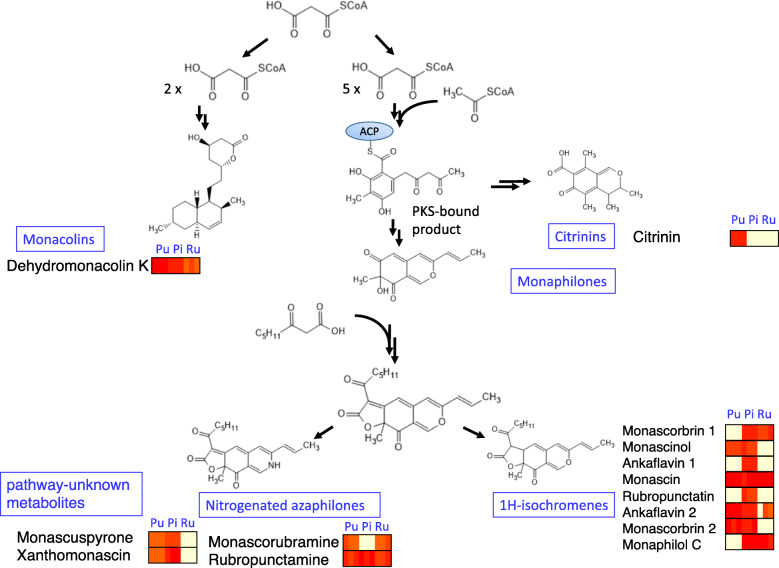
The metabolic pathways of five major groups: (i) monacolins, (ii) citrinins, (iii) monaphilines, (iv) 1H-isochromenes, and (v) nitrogenated azaphilones. The color bars represent the average concentration of metabolites in three replicates shown in Fig. 1b

In order to understand the difference of the metabolites produced in these three species, we investigated their biosynthesis pathways in detail and illustrated in Fig. 2. It has been identified that the biosynthesis of the precursor of these metabolites, 1H-isochromenes are started from malonyl-CoA, in *Penicillium marneffei* and *M. ruber* [17, 18]. Citrinin polyketide synthase (PKS) converts the PKS-bound product citrinin [17]. The biosynthetic pathway from malonyl-CoA to monacolins was also determined in *Aspergillus terreus, M. ruber*, and *M. purpureus* [19, 20]. It should be noted that PKS-bound products are acted upon by two different types of PKS enzymes – one is an enzyme to produce *Monascus* azaphilone pigments, which corresponds to the pathway from malonyl-CoA to 1H-isochromenes and nitrogenated azaphilones [17], and the other is citrinin polyketide synthase, which corresponds to the pathway from PKS-bound product to citrinin [21]. We also added the amount of the metabolites associated with each precursor as color bars. Among the three *Monascus* species, all eight metabolites related to 1H-isochromenes were only detected in *M. pilosus*. As for the other pathways, while citrinin was observed only in *M. purpureus*, Dehydromonacolin K, which is a precursor for monacolin K production, was detected in all three species. These resutls showed that the production of secondary metabolites are distinct among these three *Monascus* species. Azaphilone pigments related to 1H-isochromenes and nitrogenated azaphilones were observed among the three species, but azaphilone pigments related to ankaflavin 1 and rubropunctatin were observed in *M. pilosus,* while monascorubramine was detected in the other two species. Azaphilone pigments related to ankaflavin 1 and rubropunctatin were observed in *M. pilosus,* while monascorubramine was detected in the other two species.

### Diversity and classification of secondary metabolites produced by the three *Monascus* species

**Fig. 3:**
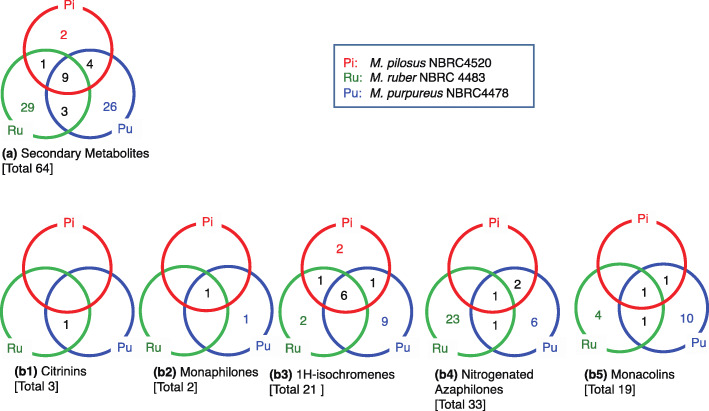
Venn diagrams of *Monascus*-specific metabolites reported by previous studies. Each Venn diagram classifies metabolies into reported species using a total of 74 previously reported secondary metabolites (a),citrinins (b1),monaphilones (b2),1H-isochromenes (b3),nitrogenated azaphilones (b4),and monacolins (b5), specific to *Monascus*-species [17, 27–30]

Fungal polyketides are the largest and most structurally diverse type of secondary metabolites, ranging from simple aromatic compounds to complex macrocyclic lactones [4]. Azaphilones are pigments with pyrone-quinone structures containing a highly oxygenated bicyclic core and a chiral quaternary center and produced mainly by *Monascus* species [21]. To examine the metabolite divergence in the three *Monascus* species, we examined the diversity of azaphilones in the abovementioned five major metabolic groups. Metabolites belonging to these groups are synthesized by PKS enzymes [19, 20]. Similar studies have been performed for 373 fungi azaphilones [4], monacolins [5, 22–25], and monaphilones [26]. We examined a total of 74 secondary metabolites (Fig. 3a) and identified the ubiquitous and species-specific metabolites in the five groups using Venn diagrams. Results showed that the three *Monascus* species can produce unique 1H-isochromene compounds. *M. ruber* and *M. purpureus* can produce a large variety of secondary metabolites, while individually, *M. pilosus*, *M. ruber,* and *M. purpureus* can produce a large variety of nitrogenated azaphilones, monacolins, and 1H-isochromenes, respectively (Fig. 3b).

### Comparison of secondary metabolite gene clusters in the three *Monascus* species

In order to measure genetic and evolutional similarity of the enzymes related in these three species, we have analyzed their whole genome sequences and annotated the sequences of the enzymes related in the biosysthesis pathways of these metabolites. Illumina MiSeq 300 bp paired-end sequencing generated 17 million high-quality short reads in individual *Monascus* species, which corresponds to 200 times the genome size. We can expect that the genome size for each species is around 24 Mb, since in the literature, the genome sizes reported for *M. purpureus* YY-1 and *M. ruber* NRRP 1597 were 24.1 Mb [12] and 24.8 Mb (https://genome.jgi.doe.gov/Monru1 /Monru1.info.html), respectively. About 94.7% of the short reads satisfied the sequencing error rate (Q20 = 1%). There were a total of 8891 genes identified in *M. purpureus*, 8645 in *M. ruber*, and 8647 in *M. pilosus*. These numbers are similar to the number of genes (7491) identified in *M. purpureus* YY-1 [12].

**Fig. 4:**
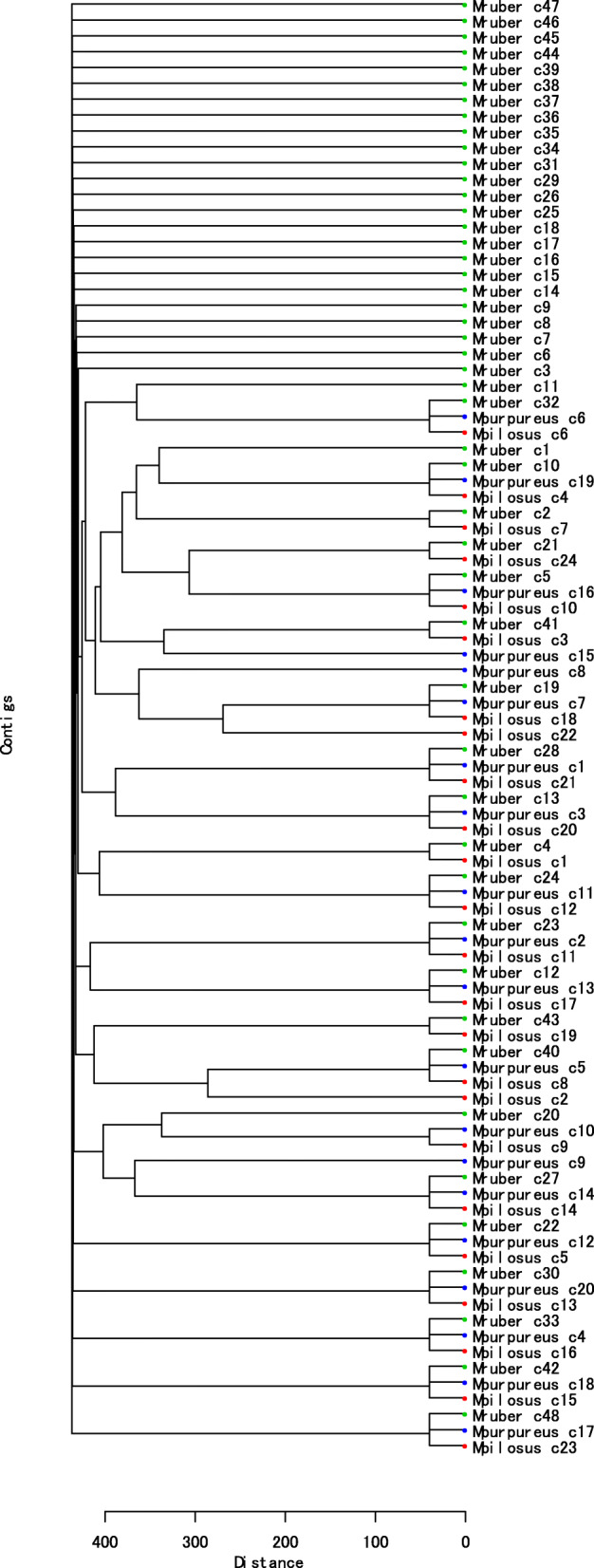
Dendrogram of secondary metabolite biosynthetic gene clusters observed in three *Monascus* species. The secondary metabolism gene clusters of *M. purpureus* NBRC 4478, *M. pilosus* NBRC 4520, and *M. ruber* NBRC 4483 estimated in this study are represented by red circles, green circles, and black circles

Furthermore, to evaluate the clusters of the enzymes related with the secondary metabolic pathways we analyzed their genome sequences using antiSMASH 4.0 [31], and we obtained 24 gene clusters for *M. pilosus*, 48 for *M. ruber,* and 20 for *M. purpureus*. Subsequently, we constructed a dendrogram using the BLAST alignment scores of the 92 gene clusters (Fig. 4), obtaining 22 groups with multiple gene clusters and 32 singletons. Gene clusters with identical gene organization were merged into a single group. The gene-coding regions were predicted using BLASTX search against UniProtKB/Swiss-Prot database (E-value < 1.0 × 10^− 10^).

**Fig. 5:**
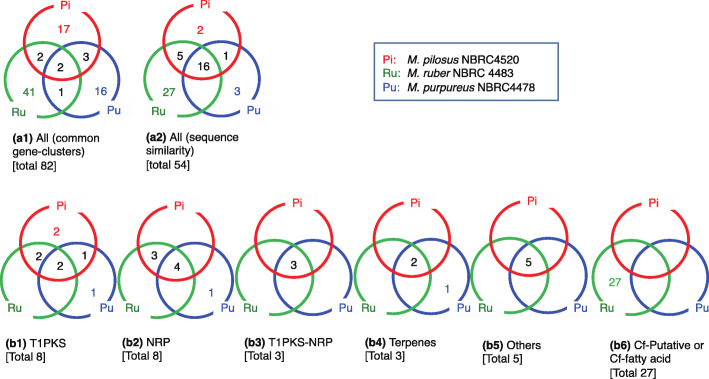
Venn diagrams of secondary metabolite biosynthetic gene clusters observed in three *Monascus* species. (a1):Venn diagram classifying 82 secondary metabolic synthesis gene clusters of three *Monascus* species. (a2):Venn diagram classifying 82 secondary metabolic synthesis gene clusters of three *Monascus* species based on DNA sequence homology. The total number of gene clusters was 54. Venn diagram classifying 54 secondary metabolic synthesis gene clusters of three *Monascus* species based on DNA sequence homology: T1PKS (b1), NRPS (b2), T1-PKS-NRPS (b3), Terpenes (b4), Others (b5), Cf-Putative or Cf-fatty acid (b6)

We also obtained 54 groups of gene clusters (Table 1) that are displayed as Venn diagrams (Fig. 5). Gene clusters were characterized as those for secondary metabolites or ABC transporters based on similarity to known genes [32]. ABC transporters are associated with PKS and NRPS gene clusters in several fungi and are responsible for the export of the corresponding secondary metabolites [33, 34].

**Table 1:** Classification of gene clusters (ID1–54) corresponding to the dendrogram in Fig. 4

ID	Type of GCs	Pi	Ru	Pu	Secondary metabolic pathways detected in DNA sequence homology	ATP-binding cassettes	Identity level
1	t1pks	1	4	–	Narbonolide/10-deoxymethynolide (*pikAI – AIV*), Phthiocerol/phenolphthionocerol (*ppsA* - *E*)	–	**
2	t1pks	2	–	–	Phthiocerol/phenolphthionocerol (*ppsA* - *E*)	–	–
3	t1pks	18	19	7	Azaphilone*, Lovastatin (LOVB, LOVF), Narbonolide/10-deoxymethynolide (*pikAI – AIV*), Phthiocerol/phenolphthionocerol (*ppsA* - *E*)	–	–
4	t1pks	22	–	–	–	–	*
5	t1pks	23	48	17	Lovastatin (LOVB, LOVF), Narbonolide/10-deoxymethynolide (*pikAI – AIV*), Phthiocerol/phenolphthionocerol (*ppsA* - *E*)	–	*
6	t1pks	19	43	–	Monacolin K*, Compactin, Lovastatin (LOVA, LOVB)	–	*
7	t1pks	–	–	8	Citrinin*, Narbonolide/10-deoxymethynolide (*pikAI–AIV*), Phthiocerol/phenolphthionocerol (*ppsA-C, E*)	–	*
8	t1pks	9	–	10	Byssochlamic acid*, Narbonolide/10-deoxymethynolide (*pikAI–AIV*), Phthiocerol/phenolphthionocerol (*ppsA-C, E*)	–	*
9	nrps	4	10	19	Neosartoricin*, Fengycin (*fenA–E*), Surfacin (*srfAA-AC*)	–	**
10	nrps	13	30	20	Brevianamide F (*FTMA*)	ABCB	**
11	nrps	7	2	–	Fengycin (*fenA-E*), Surfacin (srfAA-AC)	ABCC	**
12	nrps	11	23	2	Fengycin (*fenA-D*)	ABCC	**
13	nrps	5	22	12	Fengycin (*fenA-D*)	–	*
14	nrps	24	21	–	Fengycin (*ppsA-D*), surfacing (*srfAA-AC*)	ABCB	*
15	nrps	3	41	–	Fengycin (*fenA–D*)	–	*
16	nrps	–	–	9	Fengycin (*fenB, D*), Ferricrocin (*SIDC, SIDD*)	–	–
17	t1pks-nrps	8	40	5	NG-391*, Fengycin (*fenA–E*), Surfacin (*surAA-AC*)	ABCC	**
18	t1pks-nrps	14	27	14	Gramicidin (*grsA-B*), Fengycin (*fenA–E*), Surfacin (*surAA-AC*)	–	*
19	t1pks-nrps	20	13	3	Lovastatin (*LOVB, LOVF*), Fengycin (*fenA-E*), Surfactin (*srfAA-AC*)	ABCB	*
**ID**	**Type of GCs**	**Pi**	**Ru**	**Pu**	**Secondary metabolic pathways detected in DNA sequence homology**	**ATP-binding cassettes**	**Identity level**
20	terpene	12	24	11	Fernesyl-diphosphate (*FDFT1*)	–	**
21	terpene	17	12	13	Lupeol (*LUP1,2,4,5*), Arabidiol (*PEN1*), Tinucalladienol (*PEN3*), seco-amyrin (*PEN6*)	–	*
22	terpene	–	–	15	–	–	–
23	others	15	42	18	Fengycin (*fenA, C, E*), Surfacin (srfAA-AB)	ABCF	**
24	others	6	32	6	Kinesin (KIDFC1–3)	–	*
25	others	10	5	16	Abscisic aldehyde (AAO1–4), Fengycin (*fenA, B, E*), Surfactin (*srfAA, AB*)	ABCB	*
26	others	16	33	4	Histidinol (*hisD, IE*)	–	*
27	others	21	28	1	–	–	*
28	cf-putative	–	1	–	–	–	–
29	cf-putative	–	3	–	Palmitin (*ZDHHC*)	–	–
30	cf-putative	–	6	–	Palmitin (*ZDHHC4)*	–	–
31	cf-putative	–	7	–	–	–	–
32	cf-putative	–	8	–	–	–	–
33	cf-putative	–	9	–	–	–	–
34	cf-putative	–	11	–	–	–	–
35	cf-putative	–	14	–	Serine, Threonine (*PP1C, 2C, 4C, 6C*)	–	–
36	cf-putative	–	15	–	–	ABCG2	–
37	cf-putative	–	16	–	–	–	–
38	cf-putative	–	17	–	–	–	–
39	cf-putative	–	18	–	Mannan (*ANPI, MNN9*)	–	–
40	cf-putative	–	20	–	–	–	–
41	cf-putative	–	25	–	–	–	–
42	cf-putative	–	29	–	–	–	–
43	cf-putative	–	31	–	–	–	–
44	cf-putative	–	34	–	–	–	–
45	cf-putative	–	36	–	–	–	–
**ID**	**Type of GCs**	**Pi**	**Ru**	**Pu**	**Secondary metabolic pathways detected in DNA sequence homology**	**ATP-binding cassettes**	**Identity level**
46	cf-putative	–	37	–	–	–	–
47	cf-putative	–	38	–	–	–	–
48	cf-putative	–	39	–	Lovastatin (*LOVB-G*), Phthiocerol/Phenolphthiocerol *(ppsA,C*)	–	–
49	cf-putative	–	44	–	–	–	–
50	cf-putative	–	45	–	–	–	–
51	cf-putative	–	46	–	–	–	–
52	cf-putative	–	47	–	–	–	–
53	cf_fatty_acid	–	26	–	–	–	–
54	cf_fatty_acid	–	35	–	Fatty acid (*FAS1,2*)	–	–

The type of GCs was determined using antiSMASH software. Pi, Ru, and Pu represent the cluster ID in Fig. 4. Identical gene organization is denoted by red numbers. The secondary metabolic pathways represent the secondary metabolite information based on DNA sequence homology. Type of ATP-binding cassettes detected in individual groups is represented as ATP-binding cassettes. Gene-cluster groups with both identical gene organization and high DNA sequence similarity are denoted by ‘**’ and groups with only high DNA sequence similarity are denoted by ‘*’. Two-letter abbreviations use used for the Venn diagrams: Pi, *M. pilosus* NBRC 4520; Ru, *M. ruber* NBRC 4483; Pu, *M. purpureus* NBRC 4478

Taking gene organization in gene clusters into consideration, only two gene clusters were common in the three species. In all, 85% of the identified secondary metabolic biosynthetic gene clusters were assigned to each *Monascus* species. Thus, the organization of genes in the genome of each *Monascus* species is highly diverse (Fig. 5a1). Otherwise, if gene-clusters are classified based on DNA sequence similarity, 16 (30%) were found to be common in the three species. *M. ruber* has 27 intrinsic gene clusters, while *M. pilosus* and *M. purpureus* only have two and three intrinsic gene clusters, respectively (Fig. 5a2), indicating that the number of gene clusters in *M. ruber* is different from the other two species. Classification for individual gene clusters was conducted using antiSMASH, which identified 8 Type I polyketide biosynthetic systems (T1PKS), 8 non-ribosomal peptide biosynthesis systems (NRPS), three complex systems of T1PKS and NRPS (T1PKS-NRPS), three terpenoid biosynthetic systems, 27 putative fatty acid biosynthetic systems, and five others (Fig. 5b). The results of the present study strongly suggest that the three *Monascus* species have greatly diverged gene clusters; thus, they should be regarded as different species based on chemical taxonomy associated with the production of secondary metabolites.

We further analyzed the differences in the *Monascus* species at the DNA level by comparing the 8144-bp region where a *Monascus* azaphilone pigment biosynthetic gene cluster was localized [35]. The sequence analysis was performed for three *M. ruber* strains (NRRP 1596, JF83291.6, and NBRC 4483), two *M. purpureus* strains (NRRP 1597 and NBRC 4478), and two *M. pilosus* strains (NCBI and NBRC 4520), which identified nucleotide differences, including substitutions and insertions/deletions, at 276 bp positions (Table 2). In total, 275 positions (99.6%), except the 5194th nucleotide position, were different between the strains of (i) *M. ruber* (NRRP 1596 and NBRC 4483) and *M. pilosus* (NBRC 4520 and NCBI) and strains of (ii) *M. purpureus* (NRRP 1597 and NBRC 4478) and *M. ruber* (JF83291.6). It should be noted that NRRP 1597 and NBRC 4483 were assigned to group (i) and JF8329.6 to group (ii). *M. pilosus* is treated as a synonym of *M. ruber* in the concatenated phylogeny based on the ITS, BenA, CaM LSU, and RPB2 gene regions of 46 relative strains [36]. However, the evidence obtained in this study indicates that *M. pilosus* and *M. purpureus* are different, while *M. ruber* has similar biosynthetic gene clusters for citrinin, monacolin K, and Monascus azaphilone pigments with *M. pilosus* and *M. purpureus*. 
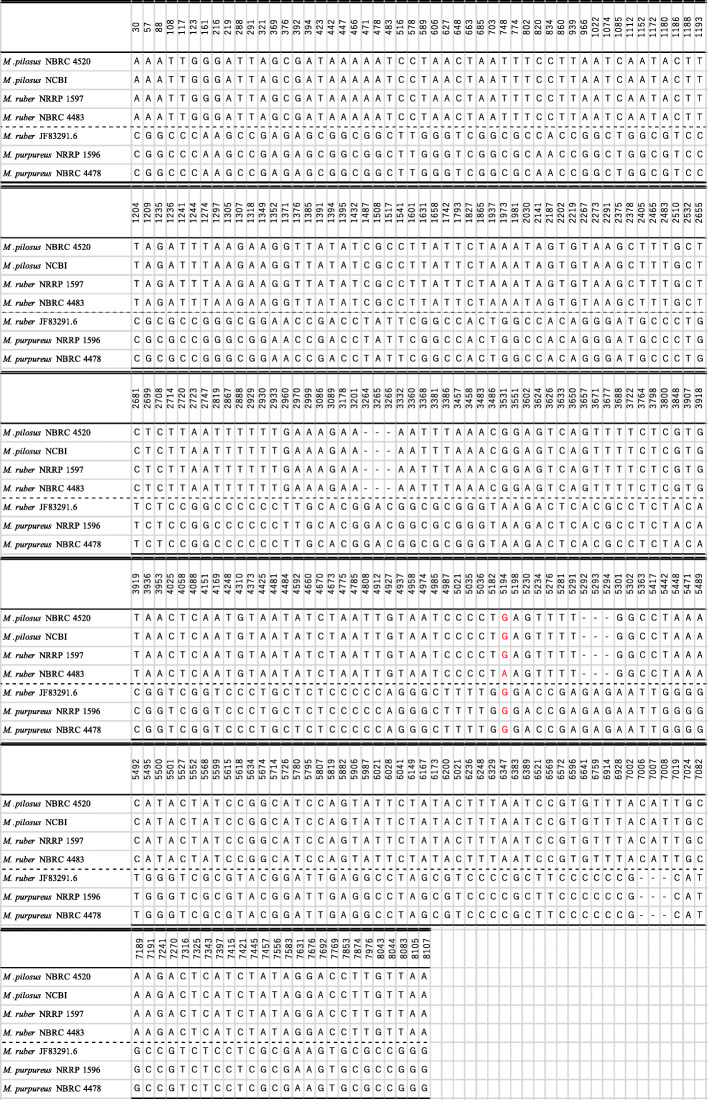

**Table 2:** Comparison of six M. purpureus, M. ruber, and M. pilosus strains based on Monascus azaphilone pigment polyketide synthase gene

	30	57	88	108	117	123	161	216	219	288	291	321	369	376	392	394	423	442	447	466	471	478	483	516	578	589	606	627	648	663	685	703	748	774	802	820	834	860	939	966	1022	1074	1085	1112	1152	1172	1180	1186	1188	1193
*M .pilosus* 4520	A	A	A	T	T	G	G	G	A	T	T	A	G	C	G	A	T	A	A	A	A	A	T	C	C	T	A	A	C	T	A	A	T	T	T	C	C	T	T	A	A	T	C	A	A	T	A	C	T	T
*M .pilosus* 4520	A	A	A	T	T	G	G	G	A	T	T	A	G	C	G	A	T	A	A	A	A	A	T	C	C	T	A	A	C	T	A	A	T	T	T	C	C	T	T	A	A	T	C	A	A	T	A	C	T	T
*M. ruber* NRLP	A	A	A	T	T	G	G	G	A	T	T	A	G	C	G	A	T	A	A	A	A	A	T	C	C	T	A	A	C	T	A	A	T	T	T	C	C	T	T	A	A	T	C	A	A	T	A	C	T	T
*M. ruber* 4483	A	A	A	T	T	G	G	G	A	T	T	A	G	C	G	A	T	A	A	A	A	A	T	C	C	T	A	A	C	T	A	A	T	T	T	C	C	T	T	A	A	T	C	A	A	T	A	C	T	T
*M. ruber* JF83291.6	C	G	G	C	C	C	A	A	G	C	C	G	A	G	A	G	C	G	G	C	G	G	C	T	T	G	G	G	T	C	G	G	C	G	C	C	A	C	C	G	G	C	T	G	G	C	G	T	C	C
*M. purpureus* v1_0	C	G	G	C	C	C	A	A	G	C	C	G	A	G	A	G	C	G	G	C	G	G	C	T	T	G	G	G	T	C	G	G	C	G	C	A	A	C	C	G	G	C	T	G	G	C	G	T	C	C
*M. purpureus* 4478	C	G	G	C	C	C	A	A	G	C	C	G	A	G	A	G	C	G	G	C	G	G	C	T	T	G	G	G	T	C	G	G	C	G	C	A	A	C	C	G	G	C	T	G	G	C	G	T	C	C
	1204	1209	1235	1236	1241	1244	1274	1297	1305	1307	1318	1349	1352	1371	1376	1385	1391	1394	1395	1432	1487	1508	1517	1541	1601	1631	1658	1742	1793	1827	1865	1937	1973	1981	2030	2141	2187	2202	2219	2267	2273	2291	2375	2378	2405	2465	2483	2510	2532	2655
*M .pilosus* 4520	T	A	G	A	T	T	T	A	A	G	A	A	G	G	T	T	A	T	A	T	C	G	C	C	T	T	A	T	T	C	T	A	A	A	T	A	G	T	G	T	A	A	G	C	T	T	T	G	C	T
*M .pilosus* 4520	T	A	G	A	T	T	T	A	A	G	A	A	G	G	T	T	A	T	A	T	C	G	C	C	T	T	A	T	T	C	T	A	A	A	T	A	G	T	G	T	A	A	G	C	T	T	T	G	C	T
*M. ruber* NRLP	T	A	G	A	T	T	T	A	A	G	A	A	G	G	T	T	A	T	A	T	C	G	C	C	T	T	A	T	T	C	T	A	A	A	T	A	G	T	G	T	A	A	G	C	T	T	T	G	C	T
*M. ruber* 4483	T	A	G	A	T	T	T	A	A	G	A	A	G	G	T	T	A	T	A	T	C	G	C	C	T	T	A	T	T	C	T	A	A	A	T	A	G	T	G	T	A	A	G	C	T	T	T	G	C	T
*M. ruber* JF83291.6	C	G	C	G	C	C	G	G	G	C	G	G	A	A	C	C	G	A	C	C	T	A	T	T	C	G	G	C	C	A	C	T	G	G	C	C	A	C	A	G	G	G	A	T	G	C	C	C	T	G
*M. purpureus* v1_0	C	G	C	G	C	C	G	G	G	C	G	G	A	A	C	C	G	A	C	C	T	A	T	T	C	G	G	C	C	A	C	T	G	G	C	C	A	C	A	G	G	G	A	T	G	C	C	C	T	G
*M. purpureus* 4478	C	G	C	G	C	C	G	G	G	C	G	G	A	A	C	C	G	A	C	C	T	A	T	T	C	G	G	C	C	A	C	T	G	G	C	C	A	C	A	G	G	G	A	T	G	C	C	C	T	G
	2681	2699	2708	2714	2720	2723	2747	2819	2867	2888	2929	2930	2933	2960	2970	2999	3086	3089	3178	3201	3264	3265	3266	3332	3360	3368	3381	3386	3457	3458	3483	3486	3531	3551	3602	3624	3626	3633	3650	3657	3671	3677	3688	3722	3764	3798	3800	3848	3907	3918
*M .pilosus* 4520	C	T	C	T	T	A	A	T	T	T	T	T	T	G	A	A	A	G	A	A	-	-	-	A	A	T	T	T	A	A	A	C	G	G	A	G	T	C	A	G	T	T	T	T	C	T	C	G	T	G
*M .pilosus* 4520	C	T	C	T	T	A	A	T	T	T	T	T	T	G	A	A	A	G	A	A	-	-	-	A	A	T	T	T	A	A	A	C	G	G	A	G	T	C	A	G	T	T	T	T	C	T	C	G	T	G
*M. ruber* NRLP	C	T	C	T	T	A	A	T	T	T	T	T	T	G	A	A	A	G	A	A	-	-	-	A	A	T	T	T	A	A	A	C	G	G	A	G	T	C	A	G	T	T	T	T	C	T	C	G	T	G
*M. ruber* 4483	C	T	C	T	T	A	A	T	T	T	T	T	T	G	A	A	A	G	A	A	-	-	-	A	A	T	T	T	A	A	A	C	G	G	A	G	T	C	A	G	T	T	T	T	C	T	C	G	T	G
*M. ruber* JF83291.6	T	C	T	C	C	G	G	C	C	C	C	C	C	T	T	G	C	A	C	G	G	A	C	G	G	C	G	C	G	G	G	T	A	A	G	A	C	T	C	A	C	G	C	C	T	C	T	A	C	A
*M. purpureus* v1_0	T	C	T	C	C	G	G	C	C	C	C	C	C	T	T	G	C	A	C	G	G	A	C	G	G	C	G	C	G	G	G	T	A	A	G	A	C	T	C	A	C	G	C	C	T	C	T	A	C	A
*M. purpureus* 4478	T	C	T	C	C	G	G	C	C	C	C	C	C	T	T	G	C	A	C	G	G	A	C	G	G	C	G	C	G	G	G	T	A	A	G	A	C	T	C	A	C	G	C	C	T	C	T	A	C	A
	3919	3936	3953	4025	4058	4088	4151	4169	4248	4310	4373	4425	4481	4484	4592	4660	4670	4673	4775	4785	4808	4912	4927	4937	4958	4974	4985	4987	5021	5035	5036	5182	5194	5198	5230	5234	5276	5281	5291	5292	5293	5294	5301	5302	5363	5417	5442	5448	5471	5489
*M .pilosus* 4520	T	A	A	C	T	C	A	A	T	G	T	A	A	T	A	T	C	T	A	A	T	T	G	T	A	A	T	C	C	C	C	T	G	A	G	T	T	T	T	-	-	-	G	G	C	C	T	A	A	A
*M .pilosus* 4520	T	A	A	C	T	C	A	A	T	G	T	A	A	T	A	T	C	T	A	A	T	T	G	T	A	A	T	C	C	C	C	T	G	A	G	T	T	T	T	-	-	-	G	G	C	C	T	A	A	A
*M. ruber* NRLP	T	A	A	C	T	C	A	A	T	G	T	A	A	T	A	T	C	T	A	A	T	T	G	T	A	A	T	C	C	C	C	T	G	A	G	T	T	T	T	-	-	-	G	G	C	C	T	A	A	A
*M. ruber* 4483	T	A	A	C	T	C	A	A	T	G	T	A	A	T	A	T	C	T	A	A	T	T	G	T	A	A	T	C	C	C	C	T	A	A	G	T	T	T	T	-	-	-	G	G	C	C	T	A	A	A
*M. ruber* JF83291.6	C	G	G	T	C	G	G	T	C	C	C	T	G	C	T	C	T	C	C	C	C	C	A	G	G	G	C	T	T	T	T	G	G	G	A	C	C	G	A	G	A	G	A	A	T	T	G	G	G	G
*M. purpureus* v1_0	C	G	G	T	C	G	G	T	C	C	C	T	G	C	T	C	T	C	C	C	C	C	A	G	G	G	C	T	T	T	T	G	G	G	A	C	C	G	A	G	A	G	A	A	T	T	G	G	G	G
*M. purpureus* 4478	C	G	G	T	C	G	G	T	C	C	C	T	G	C	T	C	T	C	C	C	C	C	A	G	G	G	C	T	T	T	T	G	G	G	A	C	C	G	A	G	A	G	A	A	T	T	G	G	G	G
	5492	5495	5500	5501	5527	5552	5568	5599	5615	5618	5634	5674	5714	5726	5780	5795	5807	5819	5882	5906	5987	6021	6028	6041	6149	6167	6173	6200	5021	6236	6248	6329	6347	6383	6389	6521	6569	6572	6596	6641	6759	6914	6928	7002	7006	7007	7008	7019	7024	7082
*M .pilosus* 4520	C	A	T	A	C	T	A	T	C	C	G	G	C	A	T	C	C	A	G	T	A	T	T	C	T	A	T	A	C	T	T	T	A	A	T	C	C	G	T	G	T	T	T	A	C	A	T	T	G	C
*M .pilosus* 4520	C	A	T	A	C	T	A	T	C	C	G	G	C	A	T	C	C	A	G	T	A	T	T	C	T	A	T	A	C	T	T	T	A	A	T	C	C	G	T	G	T	T	T	A	C	A	T	T	G	C
*M. ruber* NRLP	C	A	T	A	C	T	A	T	C	C	G	G	C	A	T	C	C	A	G	T	A	T	T	C	T	A	T	A	C	T	T	T	A	A	T	C	C	G	T	G	T	T	T	A	C	A	T	T	G	C
*M. ruber* 4483	C	A	T	A	C	T	A	T	C	C	G	G	C	A	T	C	C	A	G	T	A	T	T	C	T	A	T	A	C	T	T	T	A	A	T	C	C	G	T	G	T	T	T	A	C	A	T	T	G	C
*M. ruber* JF83291.6	T	G	G	G	T	C	G	C	G	T	A	C	G	G	A	T	T	G	A	G	G	C	C	T	A	G	C	G	T	C	C	C	C	G	C	T	T	C	C	C	C	C	C	G	-	-	-	C	A	T
*M. purpureus* v1_0	T	G	G	G	T	C	G	C	G	T	A	C	G	G	A	T	T	G	A	G	G	C	C	T	A	G	C	G	T	C	C	C	C	G	C	T	T	C	C	C	C	C	C	G	-	-	-	C	A	T
*M. purpureus* 4478	T	G	G	G	T	C	G	C	G	T	A	C	G	G	A	T	T	G	A	G	G	C	C	T	A	G	C	G	T	C	C	C	C	G	C	T	T	C	C	C	C	C	C	G	-	-	-	C	A	T
	7189	7191	7241	7270	7316	7325	7343	7397	7415	7421	7445	7457	7556	7583	7631	7676	7692	7769	7853	7874	7976	8043	8044	8083	8105	8107																								
*M .pilosus* 4520	A	A	G	A	C	T	C	A	T	C	T	A	T	A	G	G	A	C	C	T	T	G	T	T	A	A																								
*M .pilosus* 4520	A	A	G	A	C	T	C	A	T	C	T	A	T	A	G	G	A	C	C	T	T	G	T	T	A	A																								
*M. ruber* NRLP	A	A	G	A	C	T	C	A	T	C	T	A	T	A	G	G	A	C	C	T	T	G	T	T	A	A																								
*M. ruber* 4483	A	A	G	A	C	T	C	A	T	C	T	A	T	A	G	G	A	C	C	T	T	G	T	T	A	A																								
*M. ruber* JF83291.6	G	C	C	G	T	C	T	C	C	T	C	G	C	G	A	A	G	T	G	C	G	C	C	G	G	G																								
*M. purpureus* v1_0	G	C	C	G	T	C	T	C	C	T	C	G	C	G	A	A	G	T	G	C	G	C	C	G	G	G																								
*M. purpureus* 4478	G	C	C	G	T	C	T	C	C	T	C	G	C	G	A	A	G	T	G	C	G	C	C	G	G	G																								

### Comparison of citrinin biosynthetic gene clusters in the three *Monascus* species

The metabolite analysis using LC-MS shown in Fig. 1b, it was shown that citrinin was produced only by *M. purpureus*. This raises the question of whether the biosynthetic pathway of citrinin can be regulated negatively via PD culture and whether this pathway exists in *M. pilosus* and *M. ruber* genomes. Previous studies have reported that the production of citrinin by *M. ruber* can be changed by altering the medium and/or culture conditions [37, 38]. To address this issue, we compared the peptide sequences of citrinin biosynthetic genes in the three *Monascus* species with those in *M. purpureus* reported by Shimizu et al. [39] and Chen et al. [40]. There are six genes associated with the citrinin biosynthetic pathway, consisting of *pksCT* (encoding polyketide synthase, PksCT/CitS), *mpl1* (a serine hydrolase, CitA), *mpl2* (an iron II oxidase, CitB), *mpl7* (an oxidreductase, CitC), *mpl4* (encoding an aldehyde dehydrogenase, CitD), and *mpl6* (a short chain dehydrogenase, CitE). Table 3 shows the homologous regions of individual genes that aligned with the reference genes (E-value < 10^− 44^). Two proteins, CitB and CitS, were shorter in *M. ruber* and *M. pilosus* than the reference sequences. However, the same proteins were conserved in *M. purpureus* compared with the reference sequences. It suggests that the citrinin biosynthetic genes, *mpl2* (CitB), and *pksCT* (CitS) were incomplete in *M. pilosus* NBRC 4520 and *M. ruber* NBRC 4483, and consequently, citrinin production is blocked in these species.

**Table 3:** Percent identity of the amino acid sequence of citrinin biosynthetic genes in three *Monascus* species

Protein	*M. pilosus* NBRC 4520		*M. ruber* NBRC 4483		*M. purpureus* NBRC 4478		Reference peptide sequence
	length	(%)	length	(%)	length	(%)	length
CitA (*mpl1*)	249	79.6	249	79.6	312	99.7	313
CitB (*mpl2*)	229	69.6	229	69.6	328	99.7	329
CitD (*mpl4*)	458	91.4	458	91.4	500	99.8	501
CitE (*mpl6*)	231	79.1	231	79.1	284	97.3	292
CitC (*mpl7*)	532	85.5	532	85.5	618	99.4	622
CitS (*pksCT*)	1525	58.8	1525	58.8	2396	92.4	2593

Reference peptide sequence is *Monascus purpureus* citrinin biosynthesis gene clusters

(https://www.ncbi.nlm.nih.gov/nuccore/AB243687.1

### Comparison of monacolin biosynthetic gene clusters in the three *Monascus* species

Monacolin K was first isolated from the medium of *M. ruber* [41] and its biosynthesis pathway was determined, in *M. pilosus* (BCRC 387072), composed of nine enzymes that have a high level of homology with genes in the monacolin K biosynthetic gene cluster of *Aspergillus terreus* [42]. Three strains of *M. purpureus,* specifically NRRP 1596, YY-1 (an industrial strain), and KACC 42430 (a laboratory strain), lack an intact monacolin K gene cluster [43]. In the present study, we also examined the monacolin gene clusters in three *Monascus* species. As shown in Table 4, all 9 Mok genes, especially MokC, MokD, and MokF, were shorter in *M. purpureus* NBRC 4478 than *M. pilosus* NBRC 4520 and *M. ruber* NBRC 4483. Thus, *M. purpureus* NBRC 4478 also lacks the complete monacolin K gene sequence.

**Table 4:** Percent identity of the amino acid sequence of monacolin biosynthetic genes in three *Monascus* species

	*M. pilosus* NBRC 4520		*M. ruber* NBRC 4483		*M. purpureus* NBRC 4478		Reference peptide sequence
Protein	length	(%)	length	(%)	length	(%)	length
MokA	1910	100	1910	100	1772	92.8	1910
MokB	1947	100	1947	100	1501	77.1	1947
MokC	339	100	339	100	28	8.3	339
MokD	263	100	263	100	154	58.6	263
MokE	265	100	265	100	231	87.2	265
MokF	258	100	258	100	167	64.7	258
MokG	996	99.0	977	100	322	32.3	977
MokH	487	100	487	100	42	8.6	487
MokI	174	100	174	100	107	61.5	174

Reference peptide sequence is *Monascus pilosus* monacolin biosynthesis gene clusters

(https://www.ncbi.nlm.nih.gov/nuccore/DQ176595.1)

## Discussion

The three *Monascus* species examined in the present study are commonly used for food fermentation in the cuisine of East Asian countries [1–3]. Citrinin, a nephrotoxic agent, was reportedly produced in *M. purpureus* but not in *M. pilosus* [30, 44, 45]. This is corroborated by the present results from the metabolome and genome analyses revealing that citrinin biosynthetic pathways in *M. pilosus* were incomplete compared with those from *M. purpureus*. The three *Monascus* species can produce ubiquitous and species-specific pigment-related compounds (Figs. 2 and 3). Analysis of gene-organization revealed 54 greatly diverged gene clusters in the three *Monascus* species studied (Fig. 5a2). Furthermore, comparison of a 8144 bp region, in which a gene cluster of *Monascus* azaphilone synthases was localized, revealed that *M. pilosus* and *M. purpureus* can be clearly distinguished at the nucleotide level. In addition, *M. ruber* NBRC 4483 and NRRP 1597 have highly similar DNA sequences with *M. pilosus*; however, *M. ruber* JF83291.6 has highly similar DNA sequences with *M. purpureus* (Table 2). Though in some phylogenetic studies [36, 46] *M. pilosus* and *M. ruber* were not distinguished, in our analysis, their phenotypes distance clearly distinct. On the other hand, taking the pigment biosynthetic gene clusters into consideration, *M. pilosus* and *M. purpureus* should be defined as different groups. Thus, based on the findings of the present study, the *Monascus* species studied here can be classified into two groups: (i) the *M. pilosus* clade and (ii) the *M. purpureus* clade. And the results shown in Table 2 suggests that there may some *M. ruber* strains which can be related with each clade.

The mycotoxin citrinin is produced by various *Penicillium*, *Aspergillus,* and *Monascus* species [40, 44, 45]. Previously studied *M. purpureus* strains (ATCC 16365 in Java, 16379 in Taiwan, IFO 30873, and DSM 1379 by [40, 47]; YY1 by [14] can produce citrinin as a secondary metabolite. However, among the *Monascus* species, two *M. pilosus* strains (BCRC 38072 in Taiwan by [40]; NBRC 4520 in this study) cannot produce citrinin. Interestingly, several previously reported *M. ruber* strains, particularly ATCC 16246, 16378, 16366, 18199, 16371, and 18199 by Chen et al. [40], AUMC 4066 (CBS109.07) and AUMC 5705 by Moharram et al. [48], NRRP 1597 by Kwon [43], and NBRC 4483 in this study, lack citrinin production activities, but other strains, such as Tiegh by Ostry et al. [47] and ATCC 96218 by Hajjaj et al. [38] have the potential to produce this secondary metabolite. Thus, *M. ruber* can be classified into citrinin-producing and non-citrinin producing types. Based on the comparison of citrinin biosynthetic proteins, the former type might correspond to *M. purpureus* strains and the latter to *M. pilosus* strains.

In the analysis of the monacolin K gene cluster, four *M. purpureus* strains*,* specifically NRRP 1596, YY-1, KACC 42430 [43], and NBRC 4478 (in this study), lack an intact monacolin K gene cluster. By contrast, *M. pilosus* NBRC 4520 and *M. ruber* NBRC 4483 have a complete set of monacolin K gene clusters. Thus, it should be noted that *M. pilosus* NBRC 4520 and *M. ruber* NBRC 4483 can produce monacolin K but lack a complete set of citrinin biosynthetic gene clusters.

The classification of strains according to the two clade groups designated as (i) *M. pilosus* and (ii) *M. purpureus* may play an important role in the food industry and industrial field through the improved utilization of *Monascus* species. However, in view of food safety, we need to confirm whether the toxins produced by some *Monascus* strains exist in the genome or metabolome. Metabolites are generally classified into primary metabolites that are essential for growth and reproduction and secondary metabolites that are usually involved in mechanisms for ecological adaptation but are not essential to regular cellular processes. Metabolic pathways can be divided into two types: one is the general pathway shared by most fungi and the other are specialized pathways that have evolved in response to specific ecologies of certain lineages and are consequently more narrowly distributed at the taxonomic level. The citrinin pathway belongs to the former as it is present in many *Penicillium*, *Aspergillus,* and *Monascus* species [30, 44, 45]. However, the biosynthetic gene cluster of *Monascus* azaphilone pigments is limited in the *Monascus* genera. The biosynthetic process of secondary metabolites forms a cluster or non-clustered gene organization that is integral to the entire spectrum of fungal ecological strategies (e.g., saprotrophic, pathogenic, and symbiotic). Gene duplication (GD) is often implicated in the evolution of fungal metabolism (Floudas et al., 2012). A second source of metabolic gene innovation in fungi is horizontal gene transfer (HGT), which includes xenobiotic catabolism [49], toxin production [50], and degradation of plant cell walls [51]. GD and HGT were more frequently found in clustered genes than in their non-clustered counterparts [52]. In the biosynthetic gene clusters of *Monascus* azaphilone pigments and citrinin, the common trends in the strains of the three *Monascus* species are explained by the suggested *M. pilosus* and *M. purpureus* clades, whereas *M. ruber* has either *M. pilosus* or *M. purpureus* characteristics. *Monascus*-specific diverged pigments may have evolved because of GD and HGT events, resulting in the creation of clustered genes in their genomes; thus, a large number of gene clusters was observed (Table 1). Chemotaxonomy, including pigment production, is the most useful way to study the divergence of *Monascus* genera. Here, we compared the PKS responsible for the biosynthesis of azaphilone pigment from three *Monascus* species (*M. pilosus*, *M. purpureus* and *M. ruber*) and six strains. More genome sequences of *Monascus* species will need to be determined to better understand the production of secondary metabolites in these organisms.

## Conclusions

In this study, the complete genome sequences of *M. pilosus* NBRC 4520, *M. purpureus* NBRC 4478, and *M. ruber* NBRC 4483 were obtained. Three biosynthetic gene clusters, specifically monacolin K, citrinin, and azaphilone pigments that are involved in secondary metabolism, were analyzed and compared. The grouping of strains according to the two clade groups, designated as (i) *M. pilosus* and (ii) *M. purpureus*, may play an important role in the food industry and industrial field through the improved utilization of *Monascus* species. The PKS genes responsible for the biosynthesis of azaphilone pigment from the three species were compared. This genome-based analysis showed *M. ruber* could not be clearly grouped as a species. However, in view of food safety, further studies are needed to confirm whether the toxins produced by some *Monascus* strains originate from the genome and not from the metabolome.

## Methods

### Strains, culture conditions, and metabolite detection

Three *Monascus* species, specifically, *M. pilosus* NBRC 4520, *M. purpureus* NBRC 4478, and *M. ruber* NBRC 4483, were obtained from the National Institute of Technology and Evaluation in Japan. The three species were cultured in potato dextrose liquid medium at 30 °C for 7 days with 140 rpm shaking in TAITC BR-23FP. A solution of 10 mg freeze-dried PDL medium added with 1 mL methanol was sonicated for 30 min to extract secondary metabolites. The extracted metabolites were measured using a Shimadzu LCMS-8040 system (Shimadzu, Kyoto, Japan) with 300 mm ODS MonoBis columns (Kyoto Monotech Co., Ltd., Kyoto, Japan). Each metabolite was estimated based on the *m/z* of each peak, referring to the *m/z* of the metabolites previously reported in *Monascus*. We measured three replicates for each species and applied two-dimensional hierarchical clustering to visualize their similarity, using the Euclidian distance of their profiles of the observed pigments concentration as a measure of similarity score and applying the Ward method.

### Genome sequencing and assembly

We isolated genomic DNA from the three species individually and sequenced them using Illumina MiSeq paired-end libraries (300 bp read each end and 500 inserts). Approximately 8.5 million reads (around 5 Gb) for each sample were obtained and assembled using ABySS 2.0 de novo assembler [28]. We obtained 5000 to 10,000 assembled scaffolds for each samples and the N50 value of the total scaffolds were 133 Kb. The accumulated total length of the assembled contigs was 24.8 M bp, which is close to that of *M. purpureus* NRRP 1596 genome (ATCC 16365) with 23.4 Mb [12] and *M. ruber* NRRP 1597 (ATCC 13692) with 24.9 Mb [12]. The raw read sequences and the assembled contig sequences are deposited to the DNA Data Bank of Japan (DDBJ) and available under accession numbers DRX224643, DRX224644, DRX224645, respectively. To identify the gene-coding regions, the nucleotide sequence of the assembled scaffolds was annotated using DIAMOND, a high throughput BLASTX compatible sequence alignment algorithm [53]. The assembled sequences were also analyzed by BLASTed using the library of the whole UniProtKB/Swiss-Prot database [54]. Annotated genes of *M. purpureus* NRRP 1596 and *M. ruber* NRRP 1597 for were used for validation, with a cutoff E-value <1E-10. We further analyzed the genomes using antiSMASH pipeline [55] to extract the functional gene clusters such as PKS, in each *Monascus* species.

## Data Availability

Raw sequence and assembled contig data are available at the DDBJ Sequence Read Archive (https://www.ddbj.nig.ac.jp/dra/) under accession numbers DRX224643, DRX224644, DRX224645.
